# Does Daily Physical Activity Level Determine the Physical Efficiency of Children After Treatment of Leukemia?

**DOI:** 10.3390/ijerph17010307

**Published:** 2020-01-01

**Authors:** Iwona Malicka, Aleksandra Kowaluk, Marek Woźniewski

**Affiliations:** The Faculty of Physiotherapy, Wroclaw University School of Physical Education, 51-612 Wroclaw, Poland; aleksandra.kowaluk@awf.wroc.pl (A.K.); marek.wozniewski@awf.wroc.pl (M.W.)

**Keywords:** child health, leukemia, physical activity, physical fitness, physical efficiency

## Abstract

The aim of the study was to assess daily physical activity level and its influence on the physical efficiency of children after the treatment of leukemia. The study was comprised of 34 children (23 boys and 11 girls) after the treatment of acute lymphoblastic leukemia or myeloid leukemia (mean age of 11.29 ± 2.81 years, mean body height of 146.88 ± 16.11 cm, and mean body weight of 43.68 ± 13.93 kg). The mean time since treatment completion was 3.09 ± 1.80 years. The level of physical activity was assessed with the Health Behavior in School-Aged Children questionnaire (HBSC). Physical efficiency was assessed based on the palant ball throw (assessment of strength, coordination, and upper limb speed), the long jump (assessment of jumping ability, speed and coordination) and the 60 m run (assessment of speed). Measurements of motor skills were normalized, classified according to age and sex, and converted into grades. The mean values obtained in the run and the ball throw showed low pass grades in the study group. In the case of the long jump, satisfactory grades were obtained. A correlation of r = 0.512 was found between vigorous physical activity (HBSC 3) and the grade in the run. A correlation of r = −0.437 was observed between the duration of computer use in leisure time (HBSC 6) and the grade in the long jump, whereas correlations of r = −0.482 and −0.485 were noted between the number hours per week spent on games (HBSC 5) and the duration of computer use in leisure time (HBSC 6) and the grade obtained in the ball throw, respectively. In addition, different levels of physical activity and physical efficiency were demonstrated depending on the time elapsed since treatment completion. Supervised programs promoting daily physical activity should include children after the treatment of leukemia. These programs should also be aimed at improving their physical efficiency.

## 1. Introduction

Recently, a steady increase in the incidence of malignant tumors has been observed [1]. Children are also more commonly affected [2]. This partly results from the growing pollution of the environment and, thus, an increased exposure to carcinogenic factors [1,3,4,5]. At the same time, many initiatives that are undertaken to increase public awareness give beneficial results. An increase in public awareness has been observed, and an ever-increasing number of people have strived to meet the recommendations for a healthy lifestyle and preventive physical activity [6,7].

Regular physical activity is important for the proper development of children. The child’s development is accompanied by motor skills and motor abilities that determine their physical efficiency [8]. However, physical efficiency is not only related to high results in motor tests. It is also associated with an appropriate health-related approach that is manifested in physical activity. Abnormal habits acquired in childhood and a sedentary lifestyle can result in many diseases in adulthood. This is particularly important in the group of children cancer survivors who are predisposed to a higher incidence of diseases of affluence compared to their healthy peers. Such conditions include cardiovascular diseases, hypertension, type II diabetes, obesity, and osteoporosis [9].

Insufficient levels of daily physical activity are commonly observed in children who undergo cancer treatment [10]. Unfortunately, such a phenomenon also adversely affects the daily habits of children cancer survivors [11], affecting their quality of life and overall fitness [12].

Patient survival rates have increased due to the increasing effectiveness of cancer treatment, which has been achieved due to the dynamic development of medicine. Currently, more than 80% of children are permanently cured or achieve long-term remission. [13]. Leukemia is the predominant cancer disease among childhood neoplasms. This affects about 26% of all children who have been diagnosed with cancer. The effectiveness of the treatment of this disease is highest in children. An increasing number of young leukemia survivors are entering adulthood [2]. As a result, the issues related to future health of survivors and their daily physical activity and physical efficiency are of crucial importance.

A meta-analysis of randomized controlled trials (34 studies) in adult patients after the completion of the main treatment related to cancer showed that physical activity is associated with improvements in physical fitness. Physical activity is associated with significantly increased peak oxygen consumption, peak power output, distance walked in six minutes, bench press weight, leg press weight, and right handgrip strength [14].

The aim of this study was to assess daily the physical activity level and its influence on the physical efficiency of children after the treatment of leukemia. We also hypothesized that time since treatment completion could influence motor outcome.

## 2. Materials and Methods

### 2.1. Study Group

The study was comprised of 34 children (23 boys and 11 girls) after the treatment of acute lymphoblastic leukemia or myeloid leukemia (mean age of 11.29 ± 2.81 years, mean body height of 146.88 ± 16.11 cm, and mean body weight of 43.68 ± 13.93 kg). The mean time since treatment completion was 3.09 ± 1.80 years. Chemotherapy was the treatment of choice. Chemotherapy cycles were administered in the outpatient setting to all the subjects. Radiotherapy was given as adjuvant treatment in the case of 2 children. The mean treatment duration was 2.12 years. Of all of the subjects, 8 patients underwent bone marrow transplantation. No comorbidities were reported.

### 2.2. Methods

The physical activity of children was assessed using the questions from the Health Behavior in School-Aged Children (HBSC) questionnaire related to health behaviors. The questions pertained to the last seven days. We assessed the number of days per week during which the subjects performed physical activity lasting at least 60 minutes a day, defined as moderate-to-vigorous physical activity (MVPA). The estimated amount of time the subjects spent on daily physical activity was also assessed. We also evaluated the frequency and duration of significant physical effort. The concept of significant physical effort was explained to each subject and was defined as an activity that resulted in an increased heart rate, temporary shortness of breath, and increased sweating. The questionnaire also included three questions related to sedentary behaviors and the time in a sitting position in front of a computer screen or TV. The random anonymous questionnaire survey was conducted in the study group by using the traditional paper form [15].

Motor skills were also assessed. The subjects participated in three athletics disciplines, i.e., the palant ball throw, long jump and 60 m run. Each motor skill was assessed in a different discipline. Strength, coordination and upper limb speed were assessed during a palant ball throw. The long jump provided information on jumping ability, speed and coordination, and the speed of the child was assessed during the run [16]. The rules for each trial in each disciple (ball throw, long jump and run) were specified in detail and were enforced by the referees.

The throw was performed without a run-up and with a use of a light palant ball of 150 g that is used in Poland for a game similar to baseball. Each child had the possibility of 3 throws, of which the best one was selected. The field of throws was determined, and the distance markers were placed every 5 m. A measuring tape was placed in the long axis of the run-up track perpendicular to the end line. The throws were measured from the designated throw line to the point where the ball fell with an accuracy of 0.5 m.

The 30 m run up for the long jump was used. At the end of run-up, there was a 1 m zone from which the takeoff took place. Each subject started the discipline at the sign of the referee. The participants had 3 jumps, of which the best one was selected. The distance between the first mark and the takeoff point was measured. The measurements were performed with a measuring tape with an accuracy of 0.01 m.

The 60 m run was performed on the track of the athletics stadium. The participants started the run (low start position) at the sign of the referee. The results were recorded with an accuracy of 0.01 s after crossing the finish line.

The physical fitness of each child was indirectly assessed by analyzing the results in total from three disciplines in which individual motor skills were evaluated. These skills reflect the current state of the body and the ability to perform various motor tasks. In turn, physical efficiency determines the ability to perform different motor tasks depending on the level of skills, i.e., strength, speed, coordination and endurance. These criteria are analyzed by using fitness tests, which include sprinting, long jump and a palant ball throw. In addition, endurance running and medicine ball throw are included for the complete assessment. However, they were excluded from our study due to the medical condition of the cancer survivors and the lack of medical consent. 

The results obtained by children in three sports disciplines were converted into school grades in accordance with the generally accepted assessment standards according to age and sex. The grading was based on the Polish national grading system.

The study was conducted during the Lower Silesian Onco-Olympic Games of Children and Adolescents that are organized annually. The games are a sports event of which the aim is to promote physical activity and the positive influence on children who are undergoing or have already undergone the treatment of cancer. The aim of the games is to overcome stereotypical misconceptions related to the harmful influence of physical effort on children during cancer treatment. The participants compete against one another in athletic competitions based on natural forms of movement. Therefore, all measurements were made under natural conditions, and stress (being a confounding factor) was eliminated.

### 2.3. Ethics

The study was approved by the Local Bioethics Committee at the University of Physical Education in Wroclaw, Poland (consent no 7/2018).

### 2.4. Statistical Analysis

The Statistica software 10.0 (StatSoft®, Cracow, Poland) was used for statistical analysis. The normality of distribution was verified by the Shapiro–Wilk test. Student’s t-test for independent samples was used to assess the physical activity, and the Mann–Whitney U test was used to assess motor skills. Spearman’s rank correlation and Pearson’s correlation were used. The level of statistical significance was adopted at *p* < 0.05.

## 3. Results

Seven days of moderate-to-vigorous physical activity (MVPA) is generally recommended. The level of physical activity in accordance with the above recommendations was declared only by 2.94% of the subjects, whereas the most frequent answer was three days weekly (26.47%). In turn, vigorous physical activity (VPA) was most often undertaken two-to-three times weekly, and the duration was approximately one hour. The detailed data are presented in Table 1.

Sedentary behavior, including screen time (watching TV, playing computer/console games, and using electronic devices), is considered negative when its duration lasts two or more hours daily on weekdays. It was related to 32.35% of those surveyed in the case of watching TV, 49.99% in the case of computer/console games, and 35.29% for electronic devices. The comparison of the results also shows that sedentary behavior increased during weekends. The detailed data are presented in Table 2.

**Figure 1:**
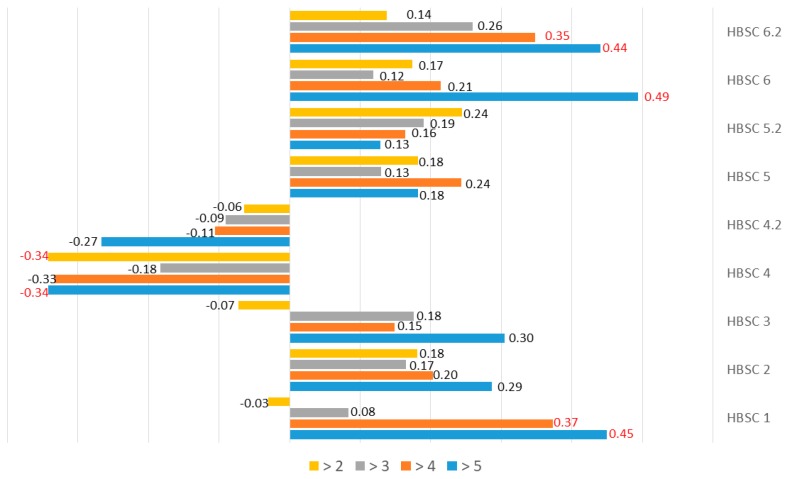
Relationships between the level of daily physical activity and the time elapsed since treatment completion. Statistically significant differences are marked in red.

Additionally, the analysis of MVPA and sedentary behavior showed a relationship with the time elapsed since treatment completion. Significant differences were demonstrated for HBSC 1, 4, 6, and 6.2 with a division into four subgroups depending on the time elapsed since treatment completion (Table 3). A positive correlation was also observed for HBSC 1, whereas a negative correlation was found for HBSC 4, 6, and 6.2. The detailed results are given in Figure 1.

After conversion into grades, the mean values obtained in the run and palant ball throw corresponded to the ‘low pass’ grade (D), and the mean value obtained in the long jump corresponded to the ‘satisfactory’ grade (C). The percentage distribution of grades in individual athletics disciplines is given in Table 4. 

**Figure 2:**
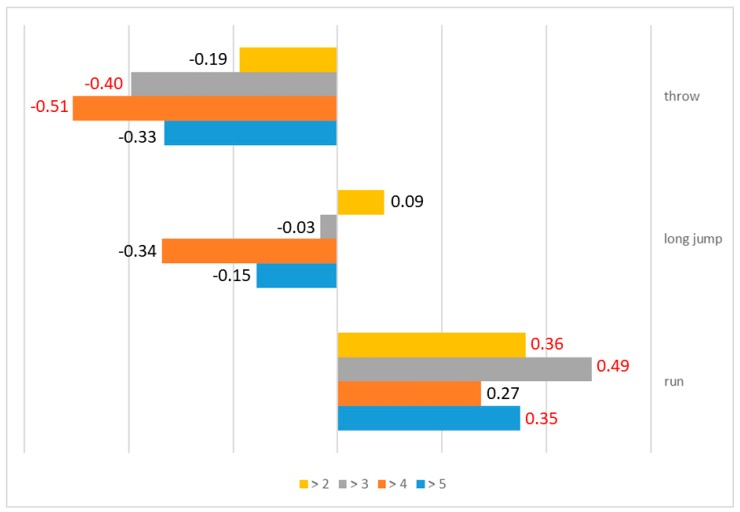
Relationships between the grades obtained in individual athletics disciplines and the time elapsed since treatment completion. Statistically significant correlations are marked in red.

The analysis of the level of individual motor skills also showed a relationship with the time elapsed since treatment completion. Significant differences were mainly observed in the case of the palant ball throw and a tendency for a change in the 60 m run when the four subgroups were considered, depending on the time elapsed since treatment completion (Table 5). Furthermore, in the case of the 60 m run, the time elapsed since treatment completion correlated positively with the grade. Therefore, the speed of the subjects improved over time. However, a negative correlation was observed in the case of the palant ball throw. Lower grades were observed for the following time frames: in the group specified as ‘To (X_1_^−^) and more than three years’ and in another group specified as ‘To (X_1_^−^) and more than four years’ after the completion of treatment, which means a shorter throw. The detailed results are given in Figure 2. 

**Figure 3:**
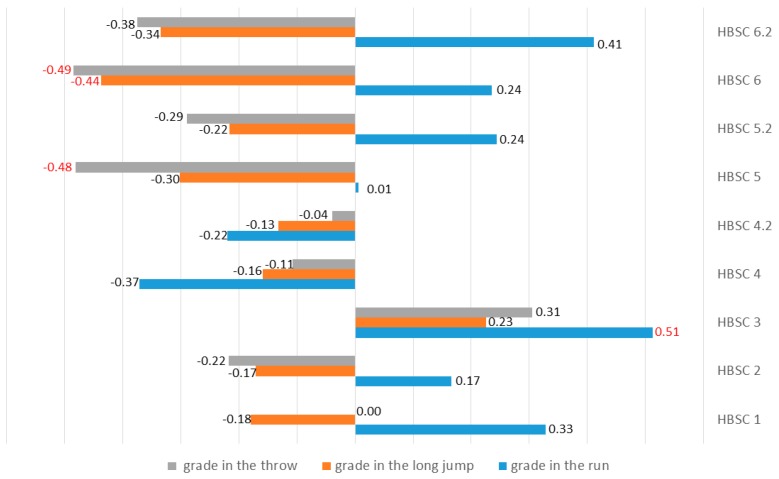
Relationships between the level of daily physical activity and the grades obtained in individual athletics disciplines. Statistically significant differences are marked in red.

A correlation of r = 0.512 was found between vigorous physical activity (HBSC 3) and the grade in the run. A correlation of r = −0.437 was observed between the duration of computer use in leisure time (HBSC 6) and the grade in the long jump, and correlations of r = −0.482 and −0.485 were found between the number hours per week spent on games (HBSC 5) and duration of computer use in leisure time (HBSC 6) and the grade obtained in the ball throw, respectively (Figure 3).

## 4. Discussion

Long-term sequelae are due to cancerous diseases and their treatment. After the treatment of leukemia, children can have persistent muscle weakness and a range of motion limitations [17]. The severity of the pathological changes depends on the child’s age, treatment specificity, and intensity. The presence and severity of such changes determines health condition and the quality of life [18].

In our study group, after the completion of cancer treatment, the subjects were characterized by a reduced level of physical fitness that was expressed by the grades obtained in individual athletics disciplines. Over 50% of our subjects received unsatisfactory grades in the 60 m run, 25% of the subjects obtained the same grades in the long jump, and approximately 33% obtained the same grades in the ball throw. Strength, gait ability and ankle flexibility are essential for motor proficiency, i.e., running, jumping, ball skills and the ball throw [17].

Patients treated for leukemia have significant lower extremity muscle weakness, and knee extension weakness is associated with the reduced walking speed [19,20]. Peripheral muscle strength and ankle dorsiflexion are also reduced in the long-term in children treated for cancer [17,21]. Ankle range of motion and balance are important factors when treating step length deficits [22]. Shorter step length, slower velocity, and decreased cadence are found during the gait of children treated for cancer [22]. Motor deficits are found frequently among survivors.

The time elapsed since treatment completion seems to be significant as well. It has been shown that after the completion of chemotherapy, neurotoxic signs and symptoms tended to disappear, and, therefore, motor performance could be expected to improve over time [23]. In the case of the 60 m run, a significant positive relationship was found between the time elapsed since treatment completion and the grade, which means that the time elapsed since treatment completion is a favorable factor in improving the speed of children. On the other hand, the opposite situation was observed in the case of the palant ball throw. A negative relationship between the time elapsed since treatment completion and the grade indicates deteriorations in strength, coordination and upper limb speed over time. It is supposed that the fine motor disturbances may be observed for a while after the therapy. Growing evidence has suggested that chemotherapeutic drugs can disrupt motor pathway development, with reports of 18%–66% of treated children displaying gross and/or fine motor difficulties. This has been reported as deficits in balance for the gross motor domain and manual dexterity for the fine motor domain. Varied results have been reported for handwriting skills [24]. 

Physical activity is an important element of behavior that affects physical fitness. Low physical activity in children after the completion of cancer treatment can additionally increase or strengthen their low physical fitness. In addition, a lack of comprehensive physical exercises can contribute to limitations related to normal development of motor skills.

The study group was characterized by an insufficient level of daily physical activity. The most common answer in the case of MVPA was three days a week. Alias et al. showed that an older age at study entry was associated with low physical activity levels. Therefore, a younger age at diagnosis and at the completion of leukemia treatment could also lead to a better recovery of overall health, an early return to school and active participation in physical education classes, thus contributing to a higher level of daily physical activity and improvement in physical fitness [25]. In our study, MVPA increased significantly with the time elapsed since treatment completion. However, the amount of leisure time devoted to watching TV decreased significantly. This could be considered a satisfactory tendency if it had not been for a simultaneous increase in time spent on computer games and computer use. This result is different from the outcome reported by Bogg et al., who (>2 years) reported higher daily physical activity and less screen time in late survivors than in early survivors. There is considerable evidence that higher levels of screen time are associated with a variety of health harms, with evidence being the strongest for adiposity, unhealthy diet, lower cardiorespiratory fitness, depressive symptoms, and quality of life [26], regardless of the level of physical activity [27]. Children with cancer are already at an increased risk for chronic diseases and a reduced quality of life. Additionally, our study showed a relationship between screen time and the obtained grades in the palant ball throw and long jump. The more time the subjects spent using the computer/console, the worse grades they obtained. Therefore, promoting healthy behavioral patterns among children and their parents is of crucial importance. Vigorous physical activity significantly correlated with the grade in the run, which indicates the importance of physical exercise in which the physical effort is so intense that it results in shortness of breath, as reported by the subjects. Based on the obtained results, it seems that training programs should be introduced to the group of children after cancer treatment. These programs should combine moderate vigorous intensity aerobic exercise with strength training to improve both aerobic fitness and body composition. As a result, these programs may determine and improve their level of physical efficiency.

The adequate level of physical activity can lead to a reduction in the long-term effects of cancer treatment that have an influence on the musculoskeletal system and, at the same time, determine physical activity. Children with leukemia are at an increased risk for low bone mineral density, and skeletal changes are found in 20%–36% of leukemia cases. In addition, 40%−56% of children gain excessive weight both during and following treatment [28].

## 5. Limitations

There are some limitations to the study design. Our results should be confirmed in a larger group of children. Additionally, physical fitness was assessed only on the basis of the analysis of the level of physical activity without consideration given to other factors such as the diet or sleep pattern of these children, which may also have an effect on their motor skills. Further studies are warranted. They should also consider other factors that influence motor skills and should be related to other cancer diseases of childhood.

## 6. Conclusions

Levels of daily physical activity and screen time are associated with the development of motor skills that affect the physical fitness of children treated for leukemia. Therefore, supervised programs promoting daily physical activity should include children after the treatment of leukemia, irrespective of the time elapsed since treatment completion. Such programs should also be aimed at improving their physical efficiency.

## Figures and Tables

**Table 1:** The level of physical activity in children. The percentage of respondents providing a given answer [%].

Question/Variable	Answer	% of Answers
The number of days per week in which the child performed the physical activity of at least 60 minutes (MVPA)—HBSC 1	0 days	2.94
	1 day	17.66
	2 days	23.53
	3 days	26.47
	4 days	11.76
	5 days	11.76
	6 days	2.94
	7 days	2.94
Frequency of vigorous physical activity—HBSC 2	daily	0
	4−6 times/week	26.47
	2−3 times/week	35.29
	once a week	29.41
	once a month	2.94
	<once a week	5.88
	Never	0
The number of hours per week devoted to vigorous physical activity—HBSC 3	none	5.88
	about 30 min	8.82
	about 1 h	29.41
	about 2 h	17.65
	about 3 h	20.59
	about 4 h	8.82
	about 5 h	2.94
	about 6 h	2.94
	about 7 h or more	2.94

**Table 2:** Time spent in front of a computer/TV screen per week. The percentage of respondents providing a given answer [%].

Question/Variable	Answer	% of Answers (Weekdays)	% of Answers (Weekends)
The number of hours in front of a TV screen per week—HBSC 4; on the weekend—HBSC 4.2	None	8.82	2.94
	about 30 min/day	17.65	2.94
	about 1 h/day	41.18	17.65
	about 2 h/day	29.41	44.12
	about 3 h/day	2.94	17.65
	about 4 h/day	0	8.82
	about 5 h/day	0	5.88
	about 6 h/day	0	0
	about 7 h or more/day	0	0
The number of hours spent playing games per week—HBSC 5; on the weekend—HBSC 5.2	None	11.76	5.88
	about 30 min/day	11.76	2.94
	about 1 h/day	26.47	8.82
	about 2 h/day	29.41	32.35
	about 3 h/day	14.71	29.41
	about 4 h/day	2.94	8.82
	about 5 h/day	0	2.94
	about 6 h/day	0	2.94
	about 7 h or more/day	2.94	5.88
The number of hours spent in front of a computer screen per week—HBSC 6; on the weekend—HBSC 6.2	None	8.82	5.88
	about 30 min/day	23.53	8.82
	about 1 h/day	32.35	23.53
	about 2 h/day	20.59	35.29
	about 3 h/day	5.88	14.71
	about 4 h/day	0	5.88
	about 5 h/day	2.94	0
	about 6 h/day	0	0
	about 7 hours or more/day	5.88	5.88

**Table 3:** Time spent on physical activity (HBSC 1 and 4) and sedentary behavior (HBSC 6 and 6.2) in relation to the time elapsed since treatment completion.

		X_1_^−^ ± SD	X_2_^−^ ± SD	*p*
HBSC 1 [days]	To (X_1_^−^) and more than 5 years (X_2_^−^)	3.58 ± 1.52	5.60 ± 0.89	0.007
	To (X_1_^−^) and more than 4 years (X_2_^−^)	3.50 ± 1.58	4.80 ± 1.31	0.02
	To (X_1_^−^) and more than 3 years (X_2_^−^)	3.73 ± 1.75	4.00 ± 1.52	0.63
	To (X_1_^−^) and more than 2 years (X_2_^−^)	4.00 ± 1.41	3.86 ± 1.66	0.86
HBSC 4 [hours]	To (X_1_^−^) and more than 5 years (X_2_^−^)	1.22 ± 0.73	0.66 ± 1.35	0.54
	To (X_1_^−^) and more than 4 years (X_2_^−^)	1.30 ± 0.77	0.76 ± 0.61	0.05
	To (X_1_^−^) and more than 3 years (X_2_^−^)	1.30 ± 0.82	1.01 ± 0.70	0.26
	To (X_1_^−^) and more than 2 years (X_2_^−^)	1.80 ± 0.83	1.02 ± 0.70	0.03
HBSC 6 [hours]	To (X_1_^−^) and more than 5 years (X_2_^−^)	1.15 ± 1.02	3.80 ± 3.11	0.0002
	To (X_1_^−^) and more than 4 years (X_2_^−^)	1.27 ± 1.11	2.19 ± 2.69	0.16
	To (X_1_^−^) and more than 3 years (X_2_^−^)	1.30 ± 1.24	1.72 ± 2.06	0.49
	To (X_1_^−^) and more than 2 years (X_2_^−^)	0.86 ± 0.77	1.65 ± 1.84	0.35
HBSC 6.2 [hours]	To (X_1_^−^) and more than 5 years (X_2_^−^)	1.72 ± 1.02	4.00 ± 3.00	0.0002
	To (X_1_^−^) and more than 4 years (X_2_^−^)	1.66 ± 0.99	3.00 ± 2.40	0.02
	To (X_1_^−^) and more than 3 years (X_2_^−^)	1.57 ± 1.00	2.43 ± 1.93	0.12
	To (X_1_^−^) and more than 2 years (X_2_^−^)	1.66 ± 1.44	2.12 ± 1.67	0.56

Statistically significant differences are marked in red. X_1_^−^ , X_2_^−^ : mean values for groups depending on the time (number of years) since the end of treatment: x_1_ ‘to’ and x_2_ ‘more than’.

**Table 4:** Percentage distribution of grades in individual athletics disciplines.

	Fail Grade (F) [%]	Low Pass Grade (D) [%]	Satisfactory Grade (C) [%]	Good Grade (B) [%]	Very Good Grade (A) [%]	Excellent Grade (A+) [%]
60 m run	56.25	9.38	3.13	6.25	9.38	15.63
Long jump	22.58	16.13	19.35	12.90	22.58	6.45
Palant ball throw	34.38	34.38	15.63	9.38	6.25	0.00

**Table 5:** Grades obtained in individual athletics disciplines in relation to the time elapsed since treatment completion.

		X_1_^−^ ± SD	X_2_^−^ ± SD	*p*
60 m run [grade]	To (X_1_^−^) and more than 5 years (X_2_^−^)	2.18 ± 1.88	4.20 ± 2.04	0.08
	To (X_1_^−^) and more than 4 years (X_2_^−^)	2.17 ± 1.92	3.33 ± 2.12	0.17
	To (X_1_^−^) and more than 3 years (X_2_^−^)	1.28 ± 0.61	3.44 ± 2.22	0.01
	To (X_1_^−^) and more than 2 years (X_2_^−^)	1.00 ± 0.00	2.77 ± 2.08	0.07
Long jump [grade]	To (X_1_^−^) and more than 5 years (X_2_^−^)	3.26 ± 1.61	2.60 ± 1.94	0,42
	To (X_1_^−^) and more than 4 years (X_2_^−^)	3.50 ± 1.47	2.33 ± 1.87	0.07
	To (X_1_^−^) and more than 3 years (X_2_^−^)	3.20 ± 1.47	3.12 ± 1.85	0.87
	To (X_1_^−^) and more than 2 years (X_2_^−^)	2.80 ± 1.48	3.23 ± 1.70	0.64
Palant ball throw [grade]	To (X_1_^−^) and more than 5 years (X_2_^−^)	2.32 ± 1.21	1.25 ± 0.50	0.08
	To (X_1_^−^) and more than 4 years (X_2_^−^)	2.52 ± 1.20	1.33 ± 0.70	0.007
	To (X_1_^−^) and more than 3 years (X_2_^−^)	2.64 ± 1.15	1.83 ± 1.15	0.03
	To (X_1_^−^) and more than 2 years (X_2_^−^)	3.00 ± 1.82	2.07 ± 1.08	0.33

Statistically significant differences are marked in red. Tendency for a change is given in bold. X_1_^−^ , X_2_^−^ : mean values for groups depending on the time (number of years) since the end of treatment: x_1_ ‘to’ and x_2_ ‘more than’.

## References

[1] Torre L.A., Bray F., Siegel R.L., Ferlay J., Lortet-Tieulent J., Jemal A. (2015). Global cancer statistics, 2012. CA Cancer J. Clin..

[2] Ward E., DeSantis C., Robbins A., Kohler B., Jemal A. (2014). Childhood and Adolescent Cancer Statistics. CA Cancer J. Clin..

[3] Boffetta P. (2006). Human cancer from environmental pollutants: The epidemiological evidence. Mutat. Res..

[4] Kampa M., Castanas E. (2008). Human health effects of air pollution. Environ. Pollut..

[5] Ghorani-Azam A., Riahi-Zanjani B., Balali-Mood M. (2016). Effects of air pollution on human health and practical measures for prevention in Iran. J. Res. Med. Sci..

[6] World Health Organization (WHO) (2003). Health and Development Trough Physical Activity and Sport.

[7] Michie S., Abraham C., Whittington C., McAteer J., Gupta S. (2009). Effective techniques in healthy eating and physical activity interventions: A meta-regression. Health Psychol..

[8] Roczniak W., Babuska-Roczniak M., Roczniak A., Roczniak R.G. (2015). Kryteria oceny rozwoju motorycznego uczniów szkół podstawowych. Med. Ogólna I Nauk. O Zdrowiu..

[9] Oeffinger K.C., Mertens A.C., Sklar C.A., Kawashima T., Hudson M.M., Meadows A.T., Friedman D.L., Marina N., Hobbie W., Kadan-Lottick N.S. (2006). Childhood Cancer Survivor Study. Chronic health conditions in adult survivors of childhood cancer. N. Engl. J. Med..

[10] Winter C., Müller C., Hoffmann C., Boos J., Rosenbaum D. (2010). Physical activity and childhood cancer. Pediatr. Blood Cancer.

[11] Murnane A., Gough K., Thompson K., Holland L., Conyers R. (2015). Adolescents and young adult cancer survivors: Exercise habits, quality of life and physical activity preferences. Support. Care Cancer.

[12] Berkman A.M., Lakoski S.G. (2015). Treatment, behavioral, and psychosocial components of cardiovascular disease risk among survivors of childhood and young adult cancer. J. Am. Heart Assoc..

[13] Shimomura Y., Baba R., Watanabe A., Horikoshi Y., Asami K., Hyakuna N., Iwai A., Matsushita T., Yamaji K., Hori T. (2011). Japanese Childhood Cancer and Leukemia Study Group (JCCLSG). Assessment of late cardiotoxicity of pirarubicin (THP) in children with acute lymphoblastic leukemia. Pediatr. Blood Cancer.

[14] Fong D.Y., Ho J.W., Hui B.P., Lee A.M., Macfarlane D.J., Leung S.S., Cerin E., Chan W.Y., Leung I.P., Lam S.H. (2012). Physical activity for cancer survivors: Meta-analysis of randomised controlled trials. BMJ.

[15] Kowaluk A., Woźniewski M., Malick A.I. (2019). Physical Activity and Quality of Life of Healthy Children and Patients with Hematological Cancers. Int. J. Environ. Res. Public Health.

[16] Malicka I., Mrowiec J., Sajkiewicz N., Siewierska K., Czajkowska M., Woźniewski M. (2019). Physical Fitness of School-Age Children after Cancer Treatment. Int. J. Environ. Res. Public Health.

[17] Tanner L.R., Hooke M.C. (2019). Improving body function and minimizing activity limitations in pediatric leukemia survivors: The lasting impact of the Stoplight Program. Pediatr. Blood Cancer.

[18] Fitch M.I. (2008). Living after cancer: Challenges in being a survivor. Can. Oncol. Nurs. J..

[19] Ness K.K., Hudson M.M., Pui C.H., Green D.M., Krull K.R., Huang T.T., Robison L.L., Morris E.B. (2012). Neuromuscular impairments in adult survivors of childhood acute lymphoblastic leukemia: Associations with physical performance and chemotherapy doses. Cancer.

[20] Ness K.K., Baker K.S., Dengel D.R., Youngren N., Sibley S., Mertens A.C., Gurney J.G. (2007). Body composition, muscle strength deficits and mobility limitations in adult survivors of childhood acute lymphoblastic leukemia. Pediatr. Blood Cancer.

[21] Hartman A., Van den Bos C., Stijnen T., Pieters R. (2008). Decrease in peripheral muscle strength and ankle dorsiflexion as long-term side effects of treatment for childhood cancer. Pediatr. Blood Cancer.

[22] Gilchrist L., Tanner L. (2016). Gait patterns in children with cancer and vincristine neuropathy. Pediatr. Phys. Ther..

[23] Hartman A., van den Bos C., Stijnen T., Pieters R. (2006). Decrease in motor performance in children with cancer is independent of the cumulative dose of vincristine. Cancer: Interdisciplinary International Journal of the American Cancer Society. Cancer.

[24] De Luca C.R., McCarthy M., Galvin J., Green J.L., Murphy A., Knight S., Williams J. (2013). Gross and fine motor skills in children treated for acute lymphoblastic leukaemia. Dev. Neurorehabil..

[25] Alias H., Nazi M., Adlina N., Lau Sie Chong D. (2019). Participation in Physical Activity and Physical Education in School Among Children with Acute Lymphoblastic Leukemia After Intensive Chemotherapy. Front. Pediatr..

[26] Domingues-Montanari S. (2017). Clinical and psychological effects of excessive screen time on children. J. Paediatr. Child Health.

[27] Bogg T.F., Shaw P.J., Cohn R.J., Wakefield C.E., Hardy L.L., Broderick C., Naumann F. (2015). Physical activity and screen-time of childhood haematopoietic stem cell transplant survivors. Acta Paediatr..

[28] White J., Flohr J.A., Winter S.S., Vener J., Feinauer L.R., Ransdell L.B. (2005). Potential benefits of physical activity for children with acute lymphoblastic leukaemia. Pediatr. Rehabil..

